# Classroom Social Climate and Resilience in Secondary Education Students

**DOI:** 10.3390/ejihpe16090122

**Published:** 2026-08-24

**Authors:** Ana María Carroza-Pacheco, Benito León-del-Barco, Carolina Bringas-Molleda

**Affiliations:** 1 Centro Asociado UNED Mérida, National University of Distance Education, 06800 Mérida, Spain; anacarroza@merida.uned.es; 2 Department of Psychology and Anthropology, University of Extremadura, 10003 Cáceres, Spain; cbringas@unex.es

**Keywords:** classroom social climate, resilience, secondary education, students, external resources, group cohesion

## Abstract

Classroom social climate has become a factor of increasing interest within the educational field due to its potential influence on students’ well-being and adjustment. The present study focused on analysing the relationship between the different dimensions of classroom social climate and school resilience in a sample of 609 Spanish secondary education students aged between 11 and 17 years. The Classroom Social Climate Scale (CSC) and the School Resilience Scale (SRS) were used as data collection instruments. The data were analysed using correlations and linear regressions to examine associations while retaining the continuous nature of the resilience scores, complemented by multinomial logistic regressions to examine the probability of belonging to relatively low, medium, or high resilience resource groups. The results showed positive and significant associations between the dimensions Relationship, Interest and Communication with Teachers (RIC) and Group Cohesion and Satisfaction (GCS), and both the Internal Resources and External Resources dimensions of resilience. Linear regression models explained 17.2% and 18.0% of the variance in Internal and External Resources, respectively, whereas multinomial logistic regression models yielded Nagelkerke R^2^ values of 0.160 and 0.190. Furthermore, the GCS dimension appeared to show a stronger predictive contribution to the different dimensions of resilience, particularly for External Resources. The findings suggest that positive peer relationships and group cohesion constitute relevant contextual factors for the development of resilience during adolescence.

## 1. Introduction

Adolescence is a developmental stage characterised by significant biological, psychological, and social changes, during which young people are particularly sensitive to the influence of the different contexts in which they develop. Among these contexts, the school environment, and particularly the social climate generated within the classroom, plays an important role in students’ well-being, adjustment, and socio-emotional development. Understanding how the different dimensions of classroom social climate are related to resilience may contribute to expanding knowledge of the contextual factors that promote adolescents’ ability to cope with adverse situations and adapt positively to them.

### 1.1. Classroom Social Climate

Classroom social climate has been identified in numerous scientific studies as one of the main determinants of students’ academic and emotional success. However, it is a complex construct, both in terms of its definition and the variety of terms used to describe it, such as *atmosphere*, *environment*, *culture*, *satisfaction*, and *context* (Pérez et al., 2010). Drawing on the subjective and individual perceptions of classroom members, these authors define the construct as “the perception that each classroom member has of its internal and daily life”. Other studies broaden this perspective by describing it as the environment created by a teacher in the classroom through the implementation of certain previously established rules, together with the way in which the teacher interacts with students and the setting in which this interaction takes place (González-Valero et al., 2021). Although closely related, classroom social climate should be distinguished from school climate. While school climate is generally understood as a broad and multidimensional construct reflecting the overall quality and character of school life (Lewno-Dumdie et al., 2020), classroom social climate refers specifically to students’ perceptions of the social and relational environment within a particular classroom. Consequently, classroom social climate may differ across classrooms within the same school, depending on factors such as teacher–student interactions and peer group dynamics. Given the objectives of the present study, classroom social climate was considered the most appropriate contextual construct.

According to Herrera-Torres et al. (2016), Gutiérrez-Primo and Sánchez-Huarcay (2022), Flores et al. (2023), and Mardones-Soto (2023), classroom climate is understood as a setting for the development of interpersonal relationships, playing a key role in shaping students’ emotions, perceptions, and attitudes towards the school environment.

In the same vein, classroom climate is closely linked to socio-emotional development and socio-affective interactions amongst members of the educational community, including students, teachers, and school leaders (Enríquez-García et al., 2022; Fierro-Suero et al., 2021; Gálvez et al., 2018; López et al., 2018; Navarro & Cantillo, 2018).

In this regard, school climate can exert both positive and negative influences depending on a range of factors, directly affecting the learning environment and the well-being of all those involved (Acosta et al., 2019; Misad et al., 2022; Tánori et al., 2022). Furthermore, it has been identified as a protective factor against bullying and victimisation (Farina, 2019; Fernández-Espínola et al., 2020; Hong et al., 2018; Mucherad et al., 2018), proving crucial in preventing academic failure, promoting student inclusion, and contributing to students’ personal development, self-perception, and self-esteem (Daily et al., 2020; Mardones-Soto, 2023; Rodríguez-Mantilla & Ruiz-Lázaro, 2019).

Moreover, a positive school climate contributes significantly to students’ intellectual development (Hackman et al., 2022) and helps to mitigate the influence of socioeconomic status on academic achievement (Berkowitz et al., 2017; Daily et al., 2020). It also promotes the development of conflict-resolution skills, encourages active participation, and strengthens the sense of belonging within the educational community (Reinoso-Molina et al., 2024). Likewise, when students’ daily experiences within educational settings are based on healthy, respectful, and cooperative relationships, the likelihood of school absenteeism decreases (Gase et al., 2017).

In contrast, studies such as that of Fierro-Suero et al. (2021) have shown that negative emotions are associated with reduced attention, lower levels of interest and motivation, and more superficial learning. In addition, Padilla and Rodríguez (2019) emphasise that a negative classroom climate has detrimental effects on educational quality, since when neither students nor teachers perceive the environment as comfortable and conducive to work, both academic and personal outcomes are compromised.

On the other hand, Barrientos et al. (2019) found that the number of students in a classroom group does not affect teachers’ ability to manage the emotional and social climate of their students. Likewise, they found no significant relationships between teachers’ ability to provide social and emotional support to their students and the creation of a suitable climate that promotes positive relationships amongst classroom members, nor with attendance at either public or state-subsidised schools.

It is important to note that school climate encompasses two dimensions: a structural dimension, which refers to the organisation of students’ roles and expectations within the classroom, as well as the implementation of collective behavioural norms; and an affective dimension, which is related to individuals’ emotional and social well-being (Toribio-Gavidia, 2025). In this context, Fierro-Suero et al. (2020) demonstrated that creating educational climates that satisfy basic psychological needs not only promotes the emergence of positive emotions but also contributes to reducing negative emotions.

Therefore, teachers play a central role, as they have the opportunity to promote resilience amongst their students by encouraging the development of their academic, physical, and social abilities within a safe and supportive environment.

### 1.2. Resilience

Resilience is broadly defined as a dynamic process that emerges following exposure to stressful, challenging, or adverse experiences, enabling individuals to adapt positively (Frias et al., 2020; Haktanir et al., 2021; Rosales-Pérez, 2023). However, conceptualisations vary: some authors describe it as a human ability that enables individuals to cope with adversity and live productively (Oducado et al., 2021), whereas others define it as a capacity associated with emotional intelligence that helps preserve well-being in the face of threats (Waugh & Sali, 2023). Although resilience has gained increasing relevance across various disciplines, no single definition has been universally accepted. Nevertheless, the prevailing view conceives resilience as a dynamic and constructive process of interactive and sociocultural origin that facilitates the optimisation of resources and positive adaptation to adversity (Kotliarenco, 2021). Within the educational context, this conceptualisation is reflected in the construct of school resilience, which constitutes the focus of the present research.

In this context, resilience is considered a multidimensional construct involving a range of both internal and external protective factors that interact to facilitate successful adaptation to challenging situations (García-Alcalá & Castro-Castañeda, 2024). In the present study, these internal and external protective factors are operationalised as resilience resources, as assessed by the School Resilience Scale. At the internal level, aspects such as self-esteem, positive self-concept, emotional self-regulation, cognitive flexibility, and the ability to establish healthy interpersonal relationships play a fundamental role (Arici-Ozcan et al., 2019; Martínez-Martí & Ruch, 2017; Rodríguez-Fernández et al., 2016; Sameroff & Rosenblum, 2006). At the external level, factors such as social support, family, school, and community environment, parenting style, as well as access to resources and opportunities, also exert a significant influence on its development (Carroza-Pacheco et al., 2025a; García-Alcalá & Castro-Castañeda, 2024; Tambo et al., 2018).

This interaction between individual and contextual factors is particularly important during adolescence, a period in which young people face a range of challenges influenced by biological, emotional, socioeconomic, and political factors, all of which affect their development both directly and indirectly (Härkönen, 2007). During this developmental stage, adolescents progressively seek greater autonomy, strengthen their identification with peer groups, and become increasingly sensitive to the quality of their interpersonal relationships, particularly within the school environment. Consequently, both internal and external resilience resources play a particularly important role in facilitating successful adaptation to the academic and social demands characteristic of this period. In this regard, resilience has been shown to predict a higher quality of life during this stage of development (Simón-Saiz et al., 2018), and may also act as a key determinant of academic achievement (Carroza-Pacheco et al., 2025b). Likewise, resilience has been identified as a moderating factor in phenomena such as cyberbullying victimisation and fatalism (Lozano & Aranzábal, 2019), thereby contributing to students’ well-being when facing both academic and social stressors. In this context, resilience enables young people to satisfy needs related to their quality of life and supports adaptability to change across different settings, constituting a fundamental component of effective emotional intelligence management at both the personal and social levels (Córdova-Gonzales et al., 2022).

Finally, various studies have identified resilience as the result of an interaction between risk and protective factors, contributing to positive adaptation, acting as a buffer against environmental difficulties, and preventing psychological maladjustment. As such, it represents an important mechanism for empowering adolescents from disadvantaged environments (Fuentes-Valdés & Saavedra-Guajardo, 2021).

### 1.3. Classroom Social Climate and Resilience

Classroom social climate was here examined through two complementary dimensions: Relationship, Interest and Communication with Teachers (RIC), which reflects the quality of teacher–student interactions and students’ interest in classroom activities, and Group Cohesion and Satisfaction (GCS), which refers to students’ perceptions of peer relationships, group cohesion, and satisfaction within the classroom.

Although studies directly exploring the relationship between classroom social climate and resilience remain limited, interest in this topic has increased in recent years. Recent studies, such as those conducted by Fenwick-Smith et al. (2018) and Gabrielli et al. (2022), emphasise that an inclusive, safe, and supportive school environment in which students perceive support, belonging, and commitment from teachers fosters conditions that strengthen resilience, although Gabrielli et al. (2022) specifically examined academic resilience among native and immigrant-origin students rather than classroom social climate. This relationship may be particularly relevant during adolescence, when increasing reliance on teachers and peers as sources of support makes the classroom social environment especially influential in the development of resilience resources.

Several studies have shown that factors such as school climate, resilience, and social support play a decisive role in preventing emotional disorders and promoting a safe and stable learning environment (Garzón, 2025). In this regard, Morcote-González and Guerrero-Arroyave (2020) found that promoting resilience in vulnerable school contexts significantly improves students’ mental health, reduces interpersonal conflicts, and decreases anxiety and stress, whilst simultaneously fostering a cooperative and empathetic environment.

Taken together, these findings suggest that relational experiences and the social context of the classroom may constitute important elements in the development of resilient resources during adolescence. However, studies specifically examining which dimensions of classroom social climate are most strongly associated with the different components of school resilience remain relatively scarce.

Although this study forms part of a broader research project that previously examined resilience and academic performance using the same sample, the present study addresses a different question, focusing specifically on the socio-educational context of the classroom. More specifically, it examines the relationship between students’ perceptions of classroom social climate and different dimensions of resilience amongst secondary education students, using the Classroom Social Climate Scale (CSC) as a specific instrument for assessing the perceived school environment.

From a theoretical perspective, these relationships may be explained by the complementary roles of teacher–student and peer interactions in fostering resilience resources. Effective communication with teachers, together with relationships characterised by trust and academic interest, may strengthen students’ internal resources, such as self-confidence, emotional regulation, and problem-solving skills. At the same time, classroom social climate is understood as a dynamic process in which students also actively contribute through their interactions with teachers and peers. Likewise, supportive peer relationships and group cohesion may reinforce external resilience resources by enhancing students’ sense of belonging, perceived social support, and access to adaptive interpersonal networks within the school context.

Unlike previous research focused on individual or family-related variables, this study adopts a contextual perspective, considering the classroom as a space of social interaction capable of influencing the development of resilient resources during adolescence. From this perspective, it is assumed that positive interpersonal relationships, perceived support, and group cohesion may particularly promote those components of resilience associated with socio-emotional adjustment and access to external support resources.

Therefore, the main objective of this study was to examine the predictive capacity of the dimensions of classroom social climate (Relationship, Interest and Communication with Teachers and Group Cohesion and Satisfaction) on the different dimensions of school resilience amongst secondary education students (Internal Resources, IRD, and External Resources, ERD).

Specifically, the following hypothesis was proposed:

**H1.**  *The different dimensions of classroom social climate (1. Relationship, Interest and Communication with Teachers and 2. Group Cohesion and Satisfaction) will be significantly associated with the resilience dimensions IRD and ERD.*

In addition to examining these associations as continuous relationships, the study also explored whether the dimensions of classroom social climate differentiated students according to their level of resilience resources, thereby providing complementary information about the probability of belonging to different resilience profiles:

**H2.**  *The different dimensions of classroom social climate (1. Relationship, Interest and Communication with Teachers and 2. Group Cohesion and Satisfaction) will be significantly associated with different levels of IRD and ERD. Specifically, it is hypothesised that higher scores in Relationship, Interest and Communication with Teachers and Group Cohesion and Satisfaction will increase the probability of belonging to high-level IRD and ERD categories, compared with low-level categories.*

## 2. Materials and Methods

### 2.1. Participants

Participants were selected using a multistage cluster sampling procedure. First, each of the 130 secondary education schools in the Extremadura region was assigned a number. Using a computerised random number generator, three schools were randomly selected. In each of the three schools, there were four classrooms per year group, and two classrooms per year group were randomly selected using the same random number procedure.

With a confidence interval of 95% and a margin of error of ±5, the sample comprised 609 secondary education students: 160 students (26.3%) from Grade 7 (aged 12–13 years), 140 (23.0%) from Grade 8 (aged 13–14 years), 158 (25.9%) from Grade 9 (aged 14–15 years), and 151 (24.8%) from Grade 10 (aged 15–16 years). Thus, the sample comprised the four years of Spanish Compulsory Secondary Education (*Educación Secundaria Obligatoria*, ESO). Of these, 305 (50.1%) were girls and 304 (49.9%) were boys. Participants ranged in age from 11 to 17 years, with a mean age of 13.43 years (SD = 1.31).

### 2.2. Materials

School Resilience Scale (SRS; Saavedra & Castro, 2009), designed to assess resilience in children aged between 9 and 14 years. It consists of 27 self-administered items rated on a 5-point Likert scale, with responses ranging from 1 (“Strongly disagree”) to 5 (“Strongly agree”). Total scores range from 27 to 135 points.

The scale comprises five dimensions, each containing a variable number of items, defined as follows:

- The Identity–Self-esteem Dimension (ISD) refers to internal strengths and the more structural aspects of personality (personal identity, self-image, and self-evaluation).
- The Networks–Models Dimension (NMD) refers to the perception of support, emotional networks, social networks, guidance, and the perception of goals.
- The Learning–Generativity Dimension (LGD) refers to opportunities for self-expression, help-seeking, coping with difficulties, and learning capacity, amongst other aspects.
- The Internal Resources Dimension (IRD) refers to the resources and conditions originating within the individual that contribute to the construction of resilient responses (e.g., “I am optimistic about the future”).
- The External Resources Dimension (ERD) refers to interactional aspects of the environment that contribute to the development of resilient behaviour (e.g., “I feel safe in the environment in which I live”).

Although the instrument was originally described as comprising five dimensions, its empirical structure is organised into three first-order factors—Identity–Self-esteem, Networks–Models, and Learning–Generativity—which in turn contribute to two second-order factors: Internal Resources and External Resources. Internal Resources comprises 13 items drawn from the three first-order factors, whereas External Resources comprises the remaining 14 items. Thus, the two second-order factors integrate items from across the three first-order factors rather than representing two additional independent sets of items. These second-order factors represent theoretically grounded composite domains that capture adolescents’ internal and contextual resilience resources and were therefore considered the most appropriate dimensions for addressing the objectives of the present study.

For our data, reliability indices were as follows: Identity–Self-esteem Dimension (ISD), Cronbach’s alpha (α = 0.78) and McDonald’s omega (Ω = 0.78); Networks–Models Dimension (NMD), Cronbach’s alpha (α = 0.83) and McDonald’s omega (Ω = 0.84); Learning–Generativity Dimension (LGD), Cronbach’s alpha (α = 0.81) and McDonald’s omega (Ω = 0.82); Internal Resources Dimension (IRD), Cronbach’s alpha (α = 0.84) and McDonald’s omega (Ω = 0.84); External Resources Dimension (ERD), Cronbach’s alpha (α = 0.89) and McDonald’s omega (Ω = 0.89); and total SRS score, Cronbach’s alpha (α = 0.91) and McDonald’s omega (Ω = 0.91). These indices demonstrate very good internal consistency.

To determine whether the structure identified by the authors of the original scale was maintained in our data, we conducted a confirmatory factor analysis using the goodness-of-fit indices presented in Table 1. As can be observed, the fit indices approach the recommended values, supporting the validity and generalisability of our results.

Classroom Social Climate Assessment Scale (CSC; Pérez et al., 2009), designed to measure classroom social climate and interpersonal interactions based on students’ perceptions of different aspects of the school environment.

It consists of 19 self-administered items rated on a 4-point Likert scale, with responses ranging from 1 (“Never/Strongly disagree”) to 4 (“Always/Strongly agree”). The scale comprises three dimensions with a variable number of items, defined as follows:

- Relationship, Interest and Communication with Teachers Dimension (RIC), referring to the level of interest, respect and communication with teachers. In essence, it assesses a positive interpersonal relationship with the teacher (e.g., “The teachers take a personal interest in all of us”).
- Group Cohesion and Satisfaction Dimension (GCS), assessing the extent to which supportive relationships, mutual knowledge and friendship are established, reflecting the quality of interpersonal relationships within the group (e.g., “We get on very well with one another in this class”).
- Peer Competitiveness Dimension (COM), assessing competitiveness in obtaining recognition, appreciation and/or rewards for achievements, as well as feelings of being more favoured in comparison with others.

Although this last dimension (Peer Competitiveness) is a factor included within the Classroom Social Climate instrument, it does not correspond to the objective of the present study, which is based on positive and favourable values for classroom coexistence. For this reason, the study focuses exclusively on the first two dimensions: Relationship, Interest and Communication with Teachers and Group Cohesion and Satisfaction.

The reliability indices for each of the two dimensions were as follows:

Relationship, Interest and Communication with Teachers Dimension (RIC), Cronbach’s alpha (α = 0.84) and McDonald’s omega (Ω = 0.84); Group Cohesion and Satisfaction Dimension (GCS), Cronbach’s alpha (α = 0.83) and McDonald’s omega (Ω = 0.84). Regarding the overall reliability of the instrument, Cronbach’s alpha was α = 0.87 and McDonald’s omega was Ω = 0.87. These indices indicate very good internal consistency.

Finally, in order to verify whether the structure identified by the authors of the Classroom Social Climate Assessment Scale was maintained in our data, a confirmatory factor analysis was conducted for two correlated factors using the goodness-of-fit indices (see Table 2). As can be observed, the fit indices showed optimal values for the two-correlated-factor model (Relationship, Interest and Communication with Teachers and Group Cohesion and Satisfaction).

### 2.3. Procedure

Data collection was carried out on the dates previously agreed with the head teachers and tutors of the schools, in the classrooms of each year group and participant group, making use of the tutorial periods scheduled in the school timetable. Each session lasted approximately one hour, including the presentation of the questionnaire and instructions for completing the booklets correctly, with particular emphasis on the need to answer all items and to do so honestly. Before completing the questionnaires, participants were instructed to answer the Classroom Social Climate Scale with reference to the general climate of their usual classroom group, rather than to any specific subject or teacher. In Spanish Compulsory Secondary Education, students generally remain with the same class group throughout the school day, while subject teachers rotate according to the timetable. Although some subjects may involve temporary regrouping, the reference group for most teaching activities is the students’ regular class group. Therefore, participants were asked to consider the overall social climate of this stable class group across subjects and teachers.

Prior to these sessions, a pilot study was conducted with a small group of secondary education students aged between 11 and 16 years in order to estimate the average time required to complete the questionnaire adequately and to verify whether the wording of the items was appropriate for their level of understanding. The results of this pilot study showed that, in all cases, the time required was less than 25 min and that any doubts regarding the wording of the items were few and could easily be resolved by the researcher and her team.

Participation was voluntary and unpaid. Informed consent was obtained beforehand from the students’ parents or legal guardians, as well as from the head teachers of the participating schools.

Despite clear instructions at the beginning of each assessment session and again when participants submitted the completed questionnaires, some questionnaires contained unanswered items. The initial sample comprised 613 students; four questionnaires (0.65%) were excluded because they contained 10% or more missing responses. Among the remaining questionnaires, 146 (23.8%) presented fewer than 10% missing items and were retained: 91 contained a single missing item, whereas the remaining 55 contained between two and six missing items. Following the standardised procedure, mean imputation based on the sample was applied to these cases. After this procedure, no further missing values remained.

### 2.4. Data Analysis

Reliability analyses (Cronbach’s alpha and McDonald’s omega) and confirmatory factor analyses were conducted for the Classroom Social Climate Assessment Scale (CSC) and the School Resilience Scale (SRS) in order to verify the adequacy of their respective theoretical structures.

Subsequently, several statistical analyses were performed according to the hypotheses:

For Hypothesis 1 (H1), Pearson’s correlation coefficient was first applied to examine the associations between the different dimensions of Classroom Social Climate and School Resilience. A linear regression analysis was then conducted to study predictive associations between the dimensions. The independent and predictor variables were the classroom social climate dimensions (1. Relationship, Interest and Communication with Teachers and 2. Group Cohesion and Satisfaction). The criterion and dependent variables were the resilience dimensions Internal Resources (IRD) and External Resources (ERD), whilst gender and age were included as control variables. Assumptions of normality (K-S, *p* > 0.05) and multicollinearity were checked prior to the analysis (VIF < 5 and Tolerance > 0.20).

For Hypothesis 2 (H2), multinomial logistic regression analyses were conducted as a complementary approach to the linear regression analyses used for H1. Whereas the linear regression analyses retained the continuous nature and full variability of the IRD and ERD scores and therefore constituted the primary analytical approach, the multinomial analyses examined how the different predictors influenced the probability of belonging to relatively low, medium, or high levels of IRD and ERD and provided odds ratios for each predictor. For these analyses, IRD and ERD scores were grouped into three levels using the 33rd and 66th percentiles as distribution-based cut-off points: low (<33rd percentile), medium (33rd–66th percentile), and high (>66th percentile). These categories represent relative sample-based groupings rather than theoretically or clinically established cut-off points.

Although cluster sampling was used (by classroom), multilevel modelling techniques were not applied because no classroom-level or contextual variables were collected, and all measures were based on students’ self-reports. Furthermore, a runs test indicated that the assumption of independence between observations was not violated (*p* = 0.10). Nevertheless, the possibility of unobserved classroom-level effects cannot be ruled out. Therefore, future research should consider the use of multilevel approaches when classroom-level variables are available, in order to account for the hierarchical structure of the data and avoid the potential overestimation of effects.

The statistical analyses were carried out using the SPSS statistical package (version 21.0 for PC) and Free JASP.

## 3. Results

Table 3 presents the descriptive statistics (means and standard deviations) for the dimensions of classroom social climate and school resilience included in the study. The subsequent analyses are presented below according to the hypotheses formulated.

### 3.1. Hypothesis H1

The first hypothesis proposed that the classroom social climate dimensions Relationship, Interest and Communication with Teachers (RIC) and Group Cohesion and Satisfaction (GCS) would be positively associated with the different components of school resilience, particularly those related to external resources and interpersonal adaptation processes.

The correlational analyses performed (Table 4) showed that both the Relationship, Interest and Communication with Teachers (RIC) dimension and the Group Cohesion and Satisfaction (GCS) dimension were positively and significantly correlated with the resilience variables analysed. Overall, the GCS dimension presented the strongest associations, particularly with External Resources, whereas the RIC dimension also showed consistent positive relationships with the resilience variables.

In order to further examine the predictive association between classroom social climate and school resilience, a series of linear regression analyses were conducted using the RIC and GCS dimensions as predictor variables and the Internal Resources (IRD) and External Resources (ERD) dimensions of resilience as dependent variables.

With regard to Internal Resources (IRD), the regression model was statistically significant (F = 32.528; *p* = 0.000), explaining 17.2% of the variance. As shown in Table 5, both the Group Cohesion and Satisfaction (GCS) dimension and the Relationship, Interest and Communication with Teachers (RIC) dimension acted as positive predictors of this variable. However, the GCS dimension showed a greater predictive weight than the RIC dimension.

With regard to External Resources (ERD), the model obtained also showed statistically significant results (F = 34.108; *p* = 0.000), explaining approximately 18.0% of the variance. Table 6 presents the coefficients corresponding to the linear regression model for this variable. Once again, both classroom social climate dimensions acted as positive predictors of resilience, although the Group Cohesion and Satisfaction (GCS) dimension showed the strongest predictive association. Overall, the results indicate that a positive relational climate and, particularly, the perception of cohesion and satisfaction among peers are associated with higher levels of school resilience in secondary education students.

### 3.2. Hypothesis H2

The second hypothesis proposed that the dimensions of classroom social climate would predict different levels of school resilience among secondary education students.

With regard to Internal Resources, the multinomial logistic regression model was statistically significant (χ^2^ = 93.505; *p* = 0.000), with a Nagelkerke value of 0.160. As shown in Table 7, the RIC and GCS dimensions contributed to differentiating the various levels of Internal Resources, with the Group Cohesion and Satisfaction dimension once again showing a greater contribution to the prediction of higher levels of resilience.

Furthermore, the odds ratio estimates derived from the model indicated that students scoring highly on the GCS dimension were 1.09 (9%) times more likely to present a medium level of Internal Resources. Likewise, students with high scores on the RIC and GCS dimensions were respectively 1.14 (14%) and 1.17 (17%) times more likely to belong to the high IRD category than to the low IRD category.

On the other hand, no significant association was found between gender or age and the different levels of IRD.

Regarding External Resources (ERD), the model obtained was also statistically significant (χ^2^ = 111.411; *p* = 0.000), reaching a Nagelkerke value of 0.190. As shown in Table 8, the classroom social climate dimensions made it possible to discriminate between the different levels of External Resources, with the Group Cohesion and Satisfaction (GCS) dimension once again emerging as the predictor with the greatest explanatory power.

With regard to the model’s odds ratio estimates, students scoring highly on the Relationship, Interest and Communication with Teachers (RIC) dimension were 1.05 (5%) times more likely to belong to the medium level of External Resources. Similarly, students scoring highly on the GCS dimension were 1.11 (11%) times more likely to belong to the medium level of External Resources. Along the same lines, students with high scores on RIC and GCS were respectively 1.15 (15%) and 1.20 (20%) times more likely to belong to the high ERD category compared with those in the low category.

As in the case of Internal Resources (IRD), no significant associations were found between gender or age and the different levels of ERD.

Overall, the results obtained from the multinomial logistic regression analyses reinforce the relationship between classroom social climate and school resilience, indicating that students who perceive higher levels of group cohesion and more positive interpersonal relationships within the classroom tend to exhibit higher levels of resilience.

## 4. Discussion

The aim of the present study was to analyse the relationship between the different dimensions of classroom social climate and school resilience in Secondary Education students, paying particular attention to the Internal Resources and External Resources dimensions of resilience. Overall, the results confirmed the hypotheses proposed, showing positive and statistically significant associations between the dimensions Relationship, Interest and Communication with Teachers (RIC) and Group Cohesion and Satisfaction (GCS), and the Internal Resources and External Resources dimensions of school resilience. These findings are consistent with previous research highlighting the importance of supportive educational environments in fostering resilience among adolescents. In particular, Romano et al. (2021) found that students’ satisfaction with relationships with teachers and classmates was associated with more favourable educational adjustment and that satisfaction with peer relationships strengthened the protective effect of academic resilience against school burnout. Likewise, although focusing on academic resilience rather than classroom social climate, Gabrielli et al. (2022) also highlighted the importance of supportive educational contexts for promoting students’ adaptive resources. Taken together, these findings reinforce the view that a positive classroom social climate constitutes an important contextual factor associated with the development of school resilience during adolescence.

More specifically, the analyses revealed that the Group Cohesion and Satisfaction (GCS) dimension showed the strongest associations with resilience, particularly with External Resources. Likewise, both the linear and multinomial logistic regression analyses indicated that this dimension maintained a greater predictive capacity than the Relationship, Interest and Communication with Teachers (RIC) dimension, even when both variables were considered simultaneously. In line with Garzón (2025), these findings seem to indicate that the perception of support, integration and satisfaction within the peer group constitutes a particularly relevant element in the development of resilient processes during adolescence.

The findings obtained are consistent with previous research highlighting the importance of relational contexts and perceived social support as protective factors against adverse situations (Martínez-Martí & Ruch, 2017; Rodríguez-Fernández et al., 2016). Similarly, several studies have shown that a positive school climate promotes emotional well-being, school adjustment and positive peer relationships (Mardones-Soto, 2023; Mucherad et al., 2018; Nickerson et al., 2019). In this regard, the results of the present study reinforce the idea that the classroom is not merely an academic setting, but also a social and emotional context that can significantly influence adolescents’ psychological adjustment. One possible explanation is that positive classroom environments provide opportunities for supportive interactions, foster students’ sense of belonging and emotional security, and facilitate the development of adaptive coping strategies through everyday relationships with teachers and peers. These contextual resources may strengthen both internal and external resilience resources by promoting confidence, perceived social support, and positive socio-emotional adjustment. Although these mechanisms were not directly examined in the present study, they offer a plausible theoretical explanation for the associations observed.

Furthermore, the apparently greater predictive weight of group cohesion compared to other dimensions of classroom social climate may be theoretically interpreted in light of the developmental characteristics of adolescence. This interpretation should be considered theoretical, as the present study did not test whether the association between GCS and resilience varied as a function of age. During adolescence, the peer group gradually acquires increasing importance in the construction of personal identity, sense of belonging, and emotional and social validation. Consequently, perceptions of integration, support and satisfaction within the peer group may act as important protective factors in the face of different situations of stress or difficulty, fostering the development of more adaptive resilient responses (Rodríguez-Fernández et al., 2016). However, this interpretation is theoretical in nature and was not directly examined in the present study, so future research should explore these mechanisms more specifically.

This interpretation is consistent with Domínguez’s (2021) proposals regarding the importance of a sense of belonging in educational settings and with Cyrulnik’s (2020) perspective, which maintains that resilient processes are fundamentally built through bonds and meaningful relationships with other people. From this perspective, resilience cannot be understood exclusively as an individual characteristic, but also as the result of the social and relational resources available within the adolescent’s immediate environment.

On the other hand, although the Relationship, Interest and Communication with Teachers (RIC) dimension showed weaker associations and predictive effects than those observed for the Group Cohesion and Satisfaction (GCS) dimension, the findings also support the relevance of teachers as protective agents within the school context. In this respect, several authors have pointed out that teacher support and the quality of teacher-student relationships contribute to the development of resilience, emotional well-being and academic adjustment (Garzón, 2025). Likewise, when such relationships deteriorate or are constrained by organisational factors or excessive workload demands, negative effects may emerge on students’ personal, emotional and academic development (Ibrahim & El Zataari, 2020).

With regard to previous research conducted within the same project, the findings obtained extend the understanding of contextual factors associated with school resilience. Whereas earlier studies examined the relationships between resilience and parenting styles, as well as between resilience and academic performance (Carroza-Pacheco et al., 2025a, 2025b), the present study incorporates classroom social climate as a relevant contextual variable. In this way, the findings suggest that both the family context and the school and relational context may play a significant role in the development of resilient processes during adolescence, which is consistent with the work of García-Alcalá and Castro-Castañeda (2024), who identify these situational environments as influential factors in the development of the capacity to adapt to adverse conditions.

Finally, the findings obtained also have potential educational and preventive implications. The promotion of positive social climates within the classroom, together with the strengthening of cooperative and supportive peer relationships, may foster not only school coexistence but also the development of personal and social resources associated with resilience. In this sense, these findings support the need for schools to assume an active role in promoting positive educational environments and strengthening the resilient resources of their students. As noted by Olmo-Extremera et al. (2021), achieving this objective requires not only interventions directed at students, but also the commitment of the entire educational community, promoting school cultures based on constructive relationships, participation and mutual support. From this perspective, programmes aimed at enhancing group cohesion, student participation and positive interpersonal relationships may contribute to the socio-emotional well-being of Secondary Education students.

### Limitations

The present study has several limitations that should be considered when interpreting the findings. First, the cross-sectional design of the research prevents the establishment of causal relationships between the variables analysed. Consequently, the results should be interpreted in terms of associations and predictive capacity rather than causality. Moreover, the observed associations may also reflect reciprocal relationships. It is possible that students with higher levels of resilience perceive classroom environments more positively or actively contribute to creating more supportive classroom climates through their own social and emotional resources. Therefore, the direction of the observed relationships cannot be established from the present data and may involve bidirectional influences.

Second, the information was obtained through self-report instruments, which may be subject to biases associated with social desirability or participants’ subjective perceptions. Furthermore, the sample was drawn from a specific geographical and educational context, which limits the generalisability of the findings to other populations or sociocultural settings.

Moreover, although this study forms part of a broader research project on school resilience, the variables analysed do not fully capture the complexity of resilient processes during adolescence. Other personal, family, social, and contextual factors may also influence the development of resilience and were not considered in the present investigation.

In addition, the categorisation of continuous IRD and ERD scores for the complementary multinomial analyses entails some loss of information, and the resulting low, medium, and high groups should be interpreted as relative sample-based categories rather than as established cut-off points.

Therefore, future research could incorporate longitudinal designs, larger and more diverse samples, as well as additional variables related to psychological well-being, school coexistence, and perceived social support, in order to further advance the understanding of the factors that promote the development of resilience during adolescence.

## 5. Conclusions

The findings of the present study allow us to conclude that classroom social climate constitutes a relevant contextual factor in the development of school resilience during adolescence. Specifically, the dimensions Relationship, Interest and Communication with Teachers (RIC) and Group Cohesion and Satisfaction (GCS) showed positive and significant associations with both the Internal Resources (IRD) and External Resources (ERD) dimensions of resilience.

Of these two dimensions, Group Cohesion and Satisfaction (GCS) emerged as the most consistent predictor of school resilience, particularly in relation to External Resources. This finding suggests the importance that peer relationships acquire during adolescence, a developmental stage in which sense of belonging, perceived social support and group integration play a fundamental role in students’ well-being and adjustment.

Furthermore, the results extend the findings obtained in previous studies conducted with the same sample by incorporating classroom social climate as a new factor associated with school resilience. In this regard, the data suggest that adolescent resilience does not depend exclusively on individual or family characteristics, but also on the relational experiences that students develop within their everyday educational context.

Taken together, these findings suggest that the promotion of positive school climates characterised by constructive interpersonal relationships, mutual support and group cohesion may contribute to strengthening the resilient resources of Secondary Education students and enhancing their personal, social and academic adjustment.

## Figures and Tables

**Table 1:** Goodness-of-fit indices of the proposed model, School Resilience Scale (SRS).

Model	χ^2^	χ^2^/df	GFI	IFI	TLI	CFI	RMSR	RMSEA
3 related factors and 2 s-order factors	1058.976	3.664	0.989	0.900	0.864	0.888	0.056	0.060

Notes: χ^2^ = chi-squared statistic; χ^2^/df = chi square divided by degrees of freedom; GFI = goodness-of-fit index; IFI = incremental fit index; TLI = Tucker–Lewis index; CFI = comparative goodness-of-fit index; RMSR = root mean square residual; RMSEA = root mean square residual of approximation.

**Table 2:** Goodness-of-fit indices of the proposed model, Classroom Social Climate Assessment Scale (CSC).

Model	χ^2^	χ^2^/df	GFI	IFI	TLI	CFI	RMSR	RMSEA
2 related factors	323.28	3.63	0.931	0.927	0.914	0.927	0.048	0.062

Notes: χ^2^ = chi-squared statistic; χ^2^/df = chi square divided by degrees of freedom; GFI = goodness-of-fit index; IFI = incremental fit index; TLI = Tucker–Lewis index; CFI = comparative goodness-of-fit index; RMSR = root mean square residual; RMSEA = root mean square residual of approximation.

**Table 3:** Means and standard deviations of the classroom social climate and school resilience dimensions.

	M	SD
RIC	23.83	4.46
GCS	21.99	4.01
IRD	53.86	7.23
ERD	60.33	8.41

Notes: M = Mean; SD = Standard Deviation; RIC = Relationship, Interest and Communication with Teachers; GCS = Group Cohesion and Satisfaction; IRD = Internal Resources Dimension; ERD = External Resources Dimension.

**Table 4:** Correlations between the dimensions of Classroom Social Climate (CSC) and the dimensions of School Resilience.

	IRD (Internal Resources)	ERD (External Resources)
RIC (Relationship, Interest and Communication with Teachers)	0.327 *	0.304 *
GCS (Group Cohesion and Satisfaction)	0.376 *	0.401 *

* *p* < 0.001.

**Table 5:** Linear regression model predicting Internal Resources in school resilience.

Model	B	SE	β	t	Sig.	Tolerance	VIF
RIC Dimension	0.340	0.068	0.209	4.986	0.000	0.773	1.294
GCS Dimension	0.503	0.075	0.279	6.701	0.000	0.787	1.270
Gender	0.920	0.539	0.064	1.707	0.088	0.979	1.022
Age	0.106	0.206	0.019	0.513	0.608	0.977	1.023

Notes: Dependent variable: Factor 4 of the School Resilience Scale (SRS), “Internal Resources” (IRD). RIC = Relationship, Interest and Communication with Teachers; GCS = Group Cohesion and Satisfaction; B = regression coefficient; β = standardised beta coefficient; SE = standard error; VIF = Variance Inflation Factor.

**Table 6:** Linear regression model predicting External Resources in school resilience.

Model	B	SE	β	t	Sig.	Tolerance	VIF
RIC Dimension	0.298	0.079	0.158	3.775	0.000	0.773	1.294
GCS Dimension	0.680	0.087	0.324	7.819	0.000	0.787	1.270
Gender	0.936	0.624	0.056	1.498	0.135	0.979	1.022
Age	−0.223	0.238	−0.035	−0.935	0.350	0.977	1.033

Notes: Dependent variable: Factor 5 of the School Resilience Scale (SRS), “External Resources” (ERD). RIC = Relationship, Interest and Communication with Teachers; GCS = Group Cohesion and Satisfaction; B = regression coefficient; β = standardised beta coefficient; SE = standard error; VIF = Variance Inflation Factor.

**Table 7:** Multinomial logistic regression model predicting levels of Internal Resources in school resilience.

Low vs. Medium vs. High Internal Resources Levels		B	SE	Wald	Sig.	Exp(B)
Medium Level of the Internal Resources Dimension (IRD) *	Participants’ gender	0.170	0.205	0.683	0.409	1.185
	Participants’ age	−0.013	0.079	0.026	0.871	0.987
	RIC Dimension	0.023	0.026	0.776	0.378	1.023
	GCS Dimension	0.087	0.028	9.734	0.002	1.091
High Level of the Internal Resources Dimension (IRD) *	Participants’ gender	0.436	0.220	3.946	0.057	1.547
	Participants’ age	0.118	0.084	1.945	0.163	1.125
	RIC Dimension	0.132	0.029	21.189	0.000	1.141
	GCS Dimension	0.159	0.032	25.009	0.000	1.173

Notes: * Low IRD level used as the reference category. Dependent variable: low, medium and high levels of Factor 4 of the School Resilience Scale (SRS), “Internal Resources” (IRD). RIC = Relationship, Interest and Communication with Teachers; GCS = Group Cohesion and Satisfaction; B = regression coefficient; SE = standard error.

**Table 8:** Multinomial logistic regression model for predicting levels of External Resources in school resilience.

Low vs. Medium vs. High External Resources Levels		B	SE	Wald	Sig.	Exp(B)
Medium Level of the External Resources Dimension (ERD) *	Participants’ gender	0.069	0.207	0.112	0.738	1.072
	Participants’ age	−0.082	0.079	1.063	0.303	0.922
	RIC Dimension	0.057	0.026	4.739	0.029	1.058
	GCS Dimension	0.109	0.029	14.573	0.000	1.115
High Level of the External Resources Dimension (ERD) *	Participants’ gender	0.241	0.220	1.201	0.273	1.273
	Participants’ age	−0.004	0.084	0.002	0.966	0.996
	RIC Dimension	0.143	0.029	24.568	0.000	1.153
	GCS Dimension	0.187	0.033	32.957	0.000	1.205

Notes: * Low ERD level used as the reference category. Dependent variable: low, medium and high levels of Factor 5 of the School Resilience Scale (SRS), “External Resources” (ERD). RIC = Relationship, Interest and Communication with Teachers; GCS = Group Cohesion and Satisfaction; B = regression coefficient; SE = standard error.

## Data Availability

The datasets generated and/or analysed during the current study are not publicly available due to ethical and legal restrictions related to the confidentiality of participant information. Access to the data is therefore limited in order to protect the privacy of the individuals involved.
